# Transport proteins determine drug sensitivity and resistance in a protozoan parasite, *Trypanosoma brucei*

**DOI:** 10.3389/fphar.2015.00032

**Published:** 2015-03-09

**Authors:** Jane C. Munday, Luca Settimo, Harry P. de Koning

**Affiliations:** ^1^Institute of Infection, Immunity and Inflammation, College of Medical, Veterinary and Life Sciences, University of GlasgowGlasgow, UK; ^2^Department of Chemistry and Chemical Biology, Northeastern UniversityBoston, MA, USA

**Keywords:** *Trypanosoma brucei*, aquaporin, aquaglyceroporin, drug transport, pentamidine, melarsoprol, drug resistance, HAPT1

## Abstract

Drug resistance in pathogenic protozoa is very often caused by changes to the ‘transportome’ of the parasites. In *Trypanosoma brucei*, several transporters have been implicated in uptake of the main classes of drugs, diamidines and melaminophenyl arsenicals. The resistance mechanism had been thought to be due to loss of a transporter known to carry both types of agents: the aminopurine transporter P2, encoded by the gene *TbAT1*. However, although loss of P2 activity is well-documented as the cause of resistance to the veterinary diamidine diminazene aceturate (DA; Berenil^®^), cross-resistance between the human-use arsenical melarsoprol and the diamidine pentamidine (melarsoprol/pentamidine cross resistance, MPXR) is the result of loss of a separate high affinity pentamidine transporter (HAPT1). A genome-wide RNAi library screen for resistance to pentamidine, published in 2012, gave the key to the genetic identity of HAPT1 by linking the phenomenon to a locus that contains the closely related *T. brucei* aquaglyceroporin genes *TbAQP2* and *TbAQP3*. Further analysis determined that knockdown of only one pore, *TbAQP2*, produced the MPXR phenotype. TbAQP2 is an unconventional aquaglyceroporin with unique residues in the “selectivity region” of the pore, and it was found that in several MPXR lab strains the WT gene was either absent or replaced by a chimeric protein, recombined with parts of *TbAQP3*. Importantly, wild-type *AQP2* was also absent in field isolates of *T. b. gambiense*, correlating with the outcome of melarsoprol treatment. Expression of a wild-type copy of *TbAQP2* in even the most resistant strain completely reversed MPXR and re-introduced HAPT1 function and transport kinetics. Expression of *TbAQP2* in *Leishmania mexicana* introduced a pentamidine transport activity indistinguishable from HAPT1. Although TbAQP2 has been shown to function as a classical aquaglyceroporin it is now clear that it is also a high affinity drug transporter, HAPT1. We discuss here a possible structural rationale for this remarkable ability.

## INTRODUCTION

African trypanosomes are extracellular parasites, which circulate in the bloodstream and tissue fluids of their mammalian hosts and are transmitted by tsetse flies in sub-Saharan Africa. They are responsible for the human disease sleeping sickness, or human African trypanosomiasis (HAT), caused by two subspecies of *Trypanosoma brucei*; *T. b. gambiense* causes the chronic form of the disease in West and Central Africa, and is responsible for the vast majority of disease cases (Brun et al., 2010), whilst *T. b. rhodesiense* causes the acute form in East Africa. Both are widely believed to be fatal unless adequate treatment is provided. The number of cases has recently decreased due to increased surveillance, treatment of cases, and targeting of the insect vector.

Both forms of HAT comprise two stages: stage one where the parasites spread through the haemo-lymphatic system from the site of the tsetse bite and stage two where the parasites cross into the cerebro-spinal fluid (CSF) via the blood-CSF and blood-brain barrier and establish an infection in the central nervous system (CNS; Mogk et al., 2014). If left untreated, the disease caused by either species leads to coma and death (Brun et al., 2010).

Most trypanosomes have to survive within two hosts, mammalian and insect, necessitating adaption to differing nutritional environments, and remodelling of their surface coat (Gadelha et al., 2011); and must also live within two specialized environments in their mammalian host. In the bloodstream and lymphatic system the parasites evade both the acquired and innate immune systems, predominantly by antigenic variation, changing the variant surface glycoprotein (VSG) expressed on their surface to avoid antibody-mediated responses (Gadelha et al., 2011). During the second stage of infection, in the CNS, they are more protected from the immune system, and may exist as a reservoir, able to reinfect the bloodstream, for example after treatment with drugs which do not penetrate into the CSF (Mogk et al., 2014).

The closely related, but human serum sensitive species *T. congolense*, *T. vivax*, and *T. brucei brucei* cause the veterinary disease animal African trypanosomiasis (AAT) or nagana, a severe, often fatal, wasting disease, principally affecting cattle, but also importantly sheep and goats, and which renders livestock farming across the tsetse belt of Africa extremely challenging (Steverding, 2008). *T. vivax* can also be transmitted mechanically by bloodsucking insects, and such transmission has been found in Central and South America (Gonzatti et al., 2014), and in non-tsetse infested regions of Ethiopia (Cherenet et al., 2006; Fikru et al., 2012). The disease Surra, a similar wasting disease to nagana, is caused by *T. evansi*, which has lost its maxicircle kinetoplast DNA and thus the procyclic stage of its lifecycle, and as such does pass through an insect vector, instead being mechanically transmitted between hosts by blood-feeding insects (Lun et al., 2010). It is the most widely distributed animal trypanosomiasis, being found in Asia, northern and northeastern Africa, Central America and South America, within a variety of host species, mainly causing disease in horses, camels, and water buffaloes, although it can also affect other equines, cattle, goats, sheep, elephants, cats, and dogs (Desquesnes et al., 2013; Namangala and Odongo, 2014). A further trypanosomiasis of horses and donkeys, dourine, is caused by the related species, *T. equiperdum*, which also cannot pass through an insect vector, being instead, uniquely amongst trypanosomal diseases, transmitted though sexual contact (Lun et al., 2010). It causes a variety of genito-urinary symptoms, along with anemia and emaciation, leading to nervous symptoms (Namangala and Odongo, 2014).

## TREATMENT OF HAT

There are currently five treatment options for HAT: pentamidine, suramin, melarsoprol, eflornithine monotherapy, and nifurtimox–eflornithine combination therapy (NECT); which drug is used is mostly dependent on disease stage and the infecting subspecies, as well as on availability of the medication. Pentamidine is used for early stage HAT caused by *T. b. gambiense*; whilst early stage *T. b. rhodesiense* is treated with suramin (Brun et al., 2010). Both were introduced in the early 20th century, with pentamidine discovered in 1937 and suramin in 1916 (Delespaux and de Koning, 2007; Steverding, 2008).

For patients with second-stage disease, melarsoprol has been widely used as the first line drug for decades, despite the fact that the drug causes fatal reactive encephalopathy in 2–5% of patients depending on the infective species (Brun et al., 2010), possibly due to rapid lysis of large amounts of parasites in the brain. Over the last 15 years, increasing rates of melarsoprol treatment failures have also been reported, with rates between 20 and 39% being seen in some foci of infections in Uganda, Republic of South Sudan, Angola, and the Democratic Republic of Congo (DRC; Legros et al., 1999; Brun et al., 2001; Moore and Richer, 2001; Stanghellini and Josenando, 2001; Robays et al., 2008; Mumba Ngoyi et al., 2010).

Eflornithine was introduced in 1990, and has been freely available since 2001 (Simarro et al., 2012), but despite the increasing failures of melarsoprol treatment, it did not replace melarsoprol as a first line treatment due to a number of significant problems: it is only effective against *T. b. gambiense* parasites, it is expensive and requires logistically difficult administration procedures, requiring four daily intravenous infusions for 14 days (Simarro et al., 2012). Partly due to the ease of inducing eflornithine resistance in the laboratory (Vincent et al., 2010) and anecdotal reports of eflornithine treatment failures, clinical trials were conducted to assess the efficacy of combinations of eflornithine, melarsoprol and the oral drug nifurtimox, which is commonly used to treat Chagas disease (Simarro et al., 2012). Ultimately, after phase III trials, the combination of nifurtimox and eflornithine (or NECT) was found to have the same safety and efficacy as eflornithine treatment alone, but to have the significant advantages of reducing both the dose and treatment time necessary for cure and of being less likely to induce resistance than eflornithine mono-therapy (Priotto et al., 2009). Thus, the World Health Organization (WHO, 2009) recommended that NECT should be used for treatment of second-stage *T. b. gambiense* sleeping sickness, and melarsoprol use has rapidly declined. Although between 2001 and 2006 the number of *T. gambiense* cases treated with eflornithine only increased from 3 to 12%, by 2009 the proportion treated was 66% and after NECT was added to the WHO essential medicines list in 2009 the proportion treated with this combination increased to 88% in 2010 (Simarro et al., 2012). Melarsoprol does of course remain the only treatment available for late-stage *T. b. rhodesiense* HAT, making study of the mechanisms of resistance crucial.

## TREATMENT OF AAT

Currently three drugs are most commonly used for AAT: the diamidine compound dimiazene aceturate (DA), and the phenanthridines isometamidium chloride (ISM) and ethidium bromide; although ISM is principally used prophylactically (Delespaux et al., 2008). The effective treatment of livestock remains hugely important for farmers within the tsetse belt of Africa, with an estimated 46 million head of cattle at risk of trypanosomiasis (Swallow, 2000). The disease is controlled by both vector control and chemotherapy, with an estimated 35 million doses of trypanocides administered annually (Geerts and Holmes, 1998), although the effectiveness of all trypanocides is threatened by drug resistance (Geerts et al., 2001; Delespaux et al., 2008).

## DRUG RESISTANCE AND TRANSPORTERS

### TBAT1/P2

Melarsoprol/pentamidine cross resistance (MPXR) is a well-known phenomenon in HAT, first described by Rollo and Williamson (1951); and although the cause was never completely resolved, it has long been clear that it was linked to reduced drug accumulation (Damper and Patton, 1976; Frommel and Balber, 1987; de Koning, 2001a). The first drug transporter identified in trypanosomes was the P2 adenosine/adenine transporter, which was originally connected to melarsoprol uptake (Carter and Fairlamb, 1993) and subsequently to diamidine transport (Barrett et al., 1995; Carter et al., 1995, 1999; de Koning and Jarvis, 2001; de Koning et al., 2004). The P2 gene was the first nucleoside transporter to be cloned from trypanosomes, with the gene designated as *TbAT1* (Mäser et al., 1999). Although the evidence for diamidine and arsenical transport by TbAT1/P2 is unquestionable, it has become equally clear that TbAT1/P2 mediates only a proportion of the uptake of both diamidines and arsenicals (de Koning, 2001b; Bray et al., 2003). The proportion of uptake varies in particular for different diamidines, as the deletion of *TbAT1* led to a high level of resistance to the veterinary diamidine DA (Matovu et al., 2003) and the newer clinical candidates furamidine and CPD0801 (Ward et al., 2011), but only to a minor loss of sensitivity for melaminophenyl arsenicals and for pentamidine (Matovu et al., 2003; Bridges et al., 2007). Thus, *T. brucei* is sensitive to these diamidines only because it expresses this unique adenosine/adenine transporter – a very rare example of a transporter with virtually equal affinity and transport efficiency for a nucleoside and its nucleobase (de Koning et al., 2005). The *TbAT1* allele may be the random result of extensive gene duplication, as the *T. brucei* genome contains at least 15 genes of the equlibrative nucleoside transporter (ENT) family (de Koning et al., 2005). Interestingly, the related parasite *T. congolense*, which is a major pathogen of livestock in sub-Saharan Africa, also has a major amplification of the ENT family (up to 19 members), but phylogenetically most of these cluster as nucleobase transporters rather than nucleoside transporters (P1-cluster) or nucleoside/nucleobase transporters (P2 cluster; Munday et al., 2013). As such, *T. congolense* does not have a counterpart of *TbAT1* (Munday et al., 2013) and is much less sensitive to diminazene (Munday and De Koning, in preparation), although this is the main drug for the treatment of *T. congolense* infection.

The mode by which TbAT1 recognizes substrates as different as diminazene, adenine, melarsoprol, adenosine and pentamidine, while displaying total selectivity for aminopurines (adenosine/adenine) over oxopurines (inosine, hypoxanthine, guanosine, guanine) has been investigated in detail. From an initial analysis of substrate selectivity, using purine analogs, it was clear that the main recognition site was the so-called ‘amidine’ motif =N-CH(R)-NH2 consisting of N1 and the 6-position amine group of the purine ring (Carter et al., 1999; de Koning and Jarvis, 1999). Substrate recognition was modelled in great detail using Comparative Molecular Field Analysis (CoMFA) and Comparative Molecular Similarity Indices analysis (CoMSIA), which produced a predictive pharmacophore model using a very diverse dataset of binding energies for 112 compounds (Collar et al., 2009).

The functional loss of TbAT1 has been linked to drug resistance in *T. brucei* species, starting with the seminal paper by Carter and Fairlamb (1993) showing that trypanosomes resistant to melaminophenyl arsenicals had lost ‘an unusual adenosine transporter.’ This transporter was cloned by Mäser et al. (1999) and a ‘resistance allele’ with several single nucleotide polymorphisms was linked to the failure to sensitize cells to the arsenicals. Similar mutations were also detected in clinical isolates and linked to high levels of melarsoprol failure in Uganda (Matovu et al., 2001). However, it has become clear that the model for melarsoprol resistance is more complicated (de Koning, 2008; Baker et al., 2013), with loss of at least one additional transporter necessary for the high-resistance phenotype (Bridges et al., 2007).

### ADDITIONAL PENTAMIDINE/ARSENICAL TRANSPORTERS IN *T. brucei*

Studies with [^125^I]-iodopentamidine showed that only half the pentamidine transport capacity of *T. brucei* is sensitive to inhibition by adenosine or adenine, and identified an additional low affinity pentamidine transporter, LAPT1 (de Koning and Jarvis, 2001). A further high affinity pentamidine transport activity, HAPT1, was identified using [^3^H]-pentamidine of high specific activity, which allowed the very low substrate concentrations required to detect this transport activity (de Koning, 2001b). This established a model of three pentamidine transporters with *K*_m_ values of approximately 35 nM (HAPT1), 300 nM (P2), and 35 μM (LAPT1; de Koning, 2008). HAPT1 was additionally found to be a transporter for the arsenical drugs, with the loss of both the TbAT1 and HAPT1 transporters simultaneously leading to high-level MPXR (Bridges et al., 2007; de Koning, 2008), and also to mediate a small proportion of diminazene uptake (Teka et al., 2011), although the latter is clinically insignificant. The very low flux of diminazene through HAPT1, relative to TbAT1, shows that this transporter is far more selective in the transport of diamidines. We have observed that, particularly, diamidines that lack a flexible linker chain between the benzamidine end groups tend to be poorly recognized and transported by HAPT1 (Ward et al., 2011), which helps explain the much higher activity of pentamidine than diminazene against *T. brucei* species.

### EFLORNITHINE TRANSPORTER

Transporters were similarly found to be crucial for sensitivity and resistance to another essential anti-trypanosomal drug, eflornithine. The *T. brucei* transporter of eflornithine, AAT6, was identified in 2010 via metabolomic analysis of eflornithine-resistant parasites, which indicated a low level of eflornithine in the resistant parasites, and the subsequent sequencing of the predicted *T. brucei* amino acid transporter genes, which found that the locus of TbAAT6 and several adjacent genes was lost in the resistant line. The identification of TbAAT6 as the transporter of eflornithine was confirmed using specific gene knockdown with RNA-interference (RNAi), which resulted in resistance to eflornithine. Moreover, the expression of TbAAT6 in an eflornithine-resistant line reversed the resistance phenotype (Vincent et al., 2010). Two RNAi library screens also identified TbAAT6 as the transporter of eflornithine and the main determinant of resistance to the drug (Baker et al., 2011; Schumann Burkard et al., 2011). TbAAT6 was subsequently confirmed to be a functional amino acid transporter, with its expression allowing growth of *Saccharomyces cerevisiae* mutants on neutral amino acids (Mathieu et al., 2014).

## AQUAPORINS IN *T. brucei*

There are three aquaglyceroporins in the *T. brucei* genome, AQP1-3, which transport a number of traditional aquaglyceroporin substrates, including water, glycerol, urea, dihydroacetone (Uzcátegui et al., 2004), and ammonia (Zeuthen et al., 2006), as well as the trivalent metalloids arsenite and antimonite (Uzcátegui et al., 2013). The relatively high number of AQPs expressed in *T. brucei* and other extracellular parasites has been hypothesized to be due to their need to survive in the extracellular environment, and/or to the differentiation between the different parasitic lifecycle stages necessary for survival in both mammalian and sandfly hosts, with the accompanying morphological and functional changes to their surface membranes (Song et al., 2014). The three *T. brucei* aquaporins have differing localisations, with TbAQP1 located on the flagellar membrane, TbAQP3 on the plasma membrane (Bassarak et al., 2011) and TbAQP2 in the flagellar pocket in bloodstream form parasites and on the plasma membrane in procyclic-form parasites (Baker et al., 2012). Interestingly, TbAQP2 and TbAQP3 are not essential for *in vivo* replication, nor indeed for testse-mediated transmission, as *in vivo* drug pressure can lead to the loss of these genes (see below).

## UNUSUAL PORE OF TbAQP2

TbAQP2 contains non-standard motifs in key portions of the gene which are thought to determine the selectivity of the pore, whereas AQP3 has standard selectivity region amino acids. TbAQP2 is the only Major Intrinsic Protein (MIP) family member so far described that has NSA/NPS and IVLL motifs, whilst TbAQP3 and TbAQP1 both contain the classical NPA/NPA motifs present in most other family members (Baker et al., 2012), and a WGYR motif in the selectivity region common to 118 aquaglyceroporin-type pores (Baker et al., 2013). Particularly, the absence of the aromatic/arginine (ar/R) motif in AQP2 may lead to an increase in its ability to transport larger and charged molecules as has been found for mammalian aquaporin 1 (Beitz et al., 2006; Li et al., 2011; Rambow et al., 2014).

## LOSS OF TbAQP2 CAUSES MELARSOPROL-PENTAMIDINE CROSS RESISTANCE (MPXR)

*T. brucei* aquaporins 2 and 3 were initially identified as being potentially important for MPXR by an RNAi library screen. The two genes are arranged on chromosome 10 in a tandem array and share 83% sequence identity (Alsford et al., 2012). By expressing each gene separately in aqp2–aqp3 double null cells, it was shown that TbAQP2 was the determinant for pentamidine and arsenical sensitivity/resistance (Baker et al., 2012).

**FIGURE 1:**
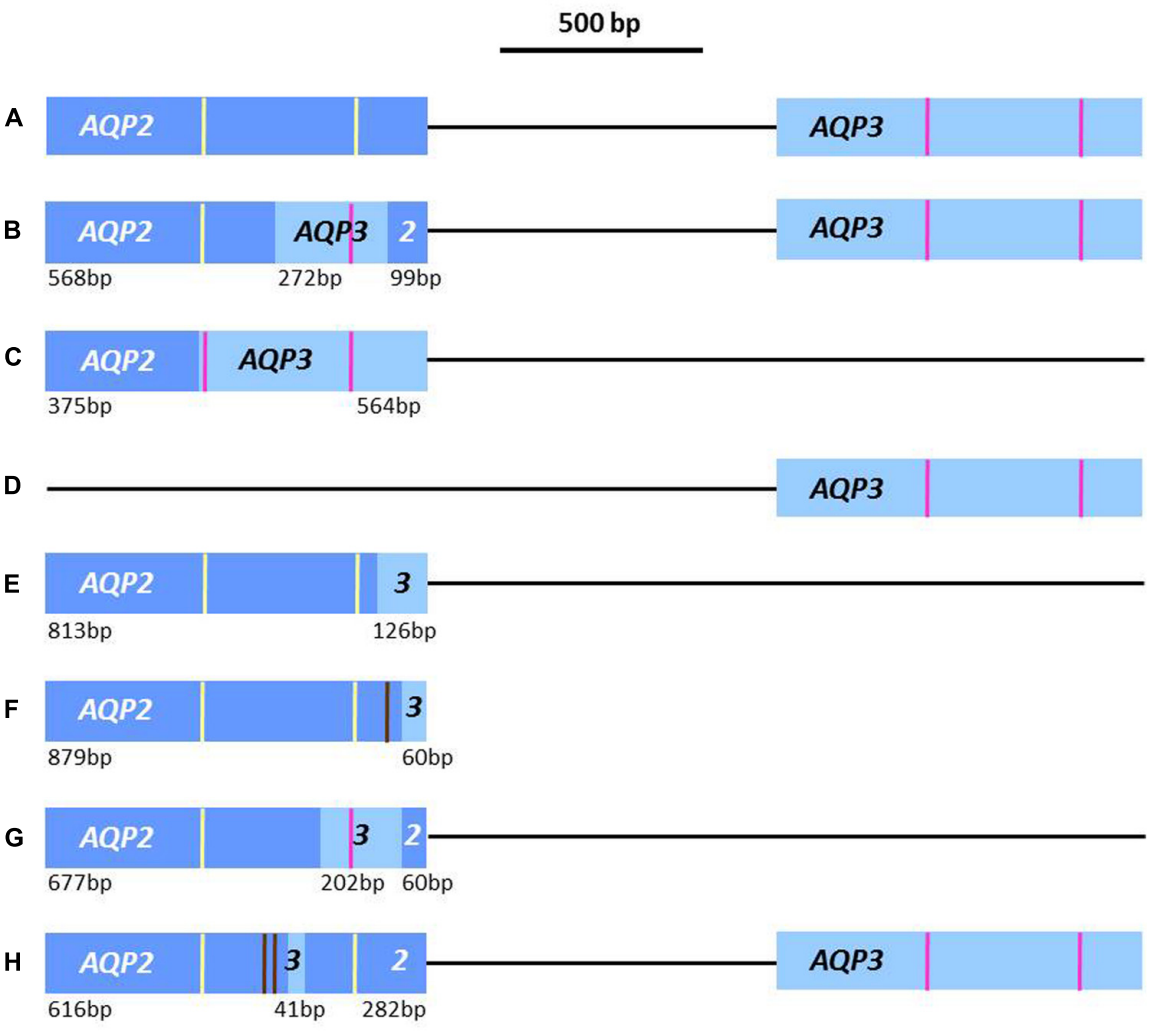
**Schematic of the documented AQP2 and AQP3 loci in lab-derived and field-isolated strains of *Trypansoma brucei*.** Brown lines = SNPs in chimeras compared to WT; yellow lines = position of NSA/NPS loci from *TbAQP2*; pink lines = position of NPA/NPA loci from *TbAQP3*. **(A)** Locus found in wildtype strains (*T. b. brucei* Lister 427, 247, and TREU927; *T. b. gambiense* 386, STIB 930, and DAL972; and *T. b. rhodesiense* STIB900 (minor differences in *TbAQP3* are not highlighted); **(B)** Locus with chimera *TbAQP2-3*_(569-841)_ from lab-derived pentamidine resistant strain B48 (Baker et al., 2012); **(C)** Locus of chimera *TbAQP2-3*_(376)_ from lab-derived melarsoprol resistant strain 247-Mr (Munday et al., 2014); **(D)** Locus in lab-derived melarsoprol resistant strains 386-Mr and STIB900-Mr; and lab-derived pentamidine resistant strain STIB900-PR (Munday et al., 2014), and in one *T. b. gambiense* K03048 allele (Graf et al., 2013); **(E)** Locus with chimera *TbAQP2-3*_(814)_ found in field isolates from Mbuji-Masi locus in DRC and the other K03048 allele (Graf et al., 2013; Pyana Pati et al., 2014); **(F)** Locus with chimera *TbAQP2-3*_(880)_ found in all *T. b. gambiense* field strains from Mbuji-Masi (Pyana Pati et al., 2014); **(G)** Locus with chimera *TbAQP2-3*_(678-880)_ found in two old Congolese *T. b. gambiense* field strains, MBA and KEMLO (Pyana Pati et al., 2014) and **(H)** Chimera *TbAQP2-3*_(617-658)_, without loss of *AQP3*, from four *T. b. gambiense* field strains isolated in Masi-Manimba (Pyana Pati et al., 2014).

The open reading frame of *TbAQP2* was investigated in the well-characterized laboratory-selected strain B48, which has high levels of resistance to both pentamidine and melarsoprol, having had *TbAT1* deleted by homologous recombination and lost HAPT1 because of continuous passage in increasing concentrations of pentamidine (Bridges et al., 2007). In this strain a *TbAQP2-3*_(569-841)_^1^ chimeric gene, inactive with respect to pentamidine sensitivity and transport, was found to have replaced *TbAQP2*; a 272-bp section toward the 3′ end of *TbAQP2* was replaced in-frame with the corresponding section of *TbAQP3* (Baker et al., 2012), see **Figures 1A,B**. This suggested that it was this latter section of the gene which is at least partially responsible for the drug-sensitivity profile of *TbAQP2*, and this section does include the second half of the likely selectivity region (NPS/IVLL is replaced with the classical regions of NPA/IGYR in the chimera). The chimeric gene was found to have assumed the location pattern of TbAQP3, being found across the whole plasma membrane (Munday et al., 2014).

The aquaporins present in other lab-derived MPXR strains, produced by selection with the water-soluble derivative of melarsoprol, cymelarsan, have also been assessed. In the *T. b. gambiense* line 386-Mr, the *TbAQP2* gene was completely absent (**Figure 1D**); whilst in the *T. b. brucei* 247-Mr line a different chimera, *TbAQP2/3*_(364)_, had been produced via the loss of both wild-type *TbAQP2* and *TbAQP3* (**Figure 1C**) The 247-Mr chimera was in frame, comprised of the first 363 bp of *TbAQP2* and the last 576 bp of *TbAQP3*; thus the protein contains both of the NPA/NPA selectivity motifs of *TbAQP3*. In a further two MPXR strains, produced by *in vivo* selection of the *T. b. rhodesiense* strain STIB900 to grow in either pentamidine or melarsoprol, the *TbAQP2* genes were found to be absent altogether (Munday et al., 2014; **Figure 1D**).

## RE-EXPRESSION OF *TbAQP2* REVERSES MPXR

Re-expression of *TbAQP2* in B48 cells re-established the sensitivity of the parasites to pentamidine and cymelarsan and restored the missing HAPT1 activity; expression of *TbAQP2* in *Leishmania mexicana* promastigotes also introduced HAPT1 function, with kinetic parameters indistinguishable from those obtained in *T. brucei*, and greatly sensitized the parasites to pentamidine and cymelarsan. In contrast, expression of the chimeric *TbAQP2-3*_(569-841)_ gene from the B48 strain in *Tbaqp2* null parasites had no significant effect on the sensitivity of the parasites to pentamidine and cymelarsan, showing that *TbAQP2-3*_(569-841)_ is not a functional pentamidine/cymelarsan transporter (Munday et al., 2014), although it is as yet unclear whether it forms a functional aquaporin.

## TbAQP2 IN FIELD ISOLATES

The above studies established that *TbAQP2* encodes the HAPT1 transport activity, and that alterations in the *TbAQP2* locus were the main determinant of MPXR in lab-derived strains. However, it was vital to verify whether TbAQP2 defects also contribute to MPXR in field isolates; this has now been completed in two separate studies, using *T. gambiense* isolates which were found to have reduced melarsoprol sensitivity in *in vivo* tests (Graf et al., 2013; Pyana Pati et al., 2014). Firstly, the *TbAQP2-AQP3* locus was genotyped in a number of *T. gambiense* field isolates (both recent and historical; from patients who either relapsed or were cured after melarsoprol treatment). In the five recent isolates from the Mbuji-Masi focus in the DRC, which were isolated from patients who relapsed after melarsoprol treatment, a single in-frame, chimeric *TbAQP2-3*_(814)_ gene was found instead of the native tandem gene locus (Graf et al., 2013). The first 813 bp of the chimera were from *TbAQP2*, with the remaining 126 bp end of *TbAQP3*. In this chimera, the putative NSA/NPS selectivity region of TbAQP2 is retained, see **Figure 1E**. In an older isolate from South Sudan, K03048, heterozygosity in the locus was observed; one allele contained only *TbAQP3*, having lost *TbAQP2*, whilst the other allele was composed of a similar *TbAQP2-3*_(814)_ chimera to that found in the DRC strains (**Figures 1D,E**). This South Sudanese isolate was also from a patient who relapsed after melarsoprol treatment. The wildtype *TbAQP2* allele was found in a number of older strains, isolated from 1960 to 1995, which came from patients either successfully cured by melarsoprol treatment, or whose treatment outcome is unknown (Graf et al., 2013).

Further recent *T. b. gambiense* isolates from the Mbuji-Masi focus in the DRC have now been analyzed, including several pairs of isolates collected before and after treatment with melarsoprol (Pyana Pati et al., 2014). In 12 of these strains, the *TbAQP2-3*_(814)_ chimera was found one of these isolates had been previously sequenced by Graf et al. (2013), which is a chimera containing only the last 43 amino acids of AQP3 (**Figure 1E**). By direct sequencing, a further *TbAQP2-3*_(880)_ chimera was found from the Mbuji-Masi strains; comprising of the first 879 bp of *TbAQP2,* with a SNP of T869C, followed by an in-frame switch to the last 60 bp of *TbAQP3* (**Figure 1F**). Thus these strains, despite being from patients both cured and relapsing after treatment with melarsoprol, seem all to contain the two heterozygous chimeric alleles, *TbAQP2-3*_(814)_ and *TbAQP2-3*_(880)_ (Pyana Pati et al., 2014). In two old isolates of *T. b. gambiense*, MBA and KEMLO (Paindavoine et al., 1986), from the DRC, another chimera was found. The patient history of these strains is unknown, however, the strains have lost the native *TbAQP2* and *TbAQP3* genes, instead having a chimera consisting of the first 677 bp of *TbAQP2*, then the next 202 bp of *TbAQP3*, followed by the final 60 bp from *TbAQP2* (see **Figure 1G**). This *TbAQP2-3*_(678-880)_ chimera, similarly to that found in the lab strain B48 (described above), lacks the second half of the likely selectivity region, with the NPS/IVLL residues of *TbAQP2* replaced with the classical region, NPA/IGYR in the chimera (Pyana Pati et al., 2014).

A further four strains of *T. b. gambiense*, isolated in 2001 from the Masi-Manimba focus in the DRC, where high relapse rates after melarsoprol treatment have never been found, were also investigated. These four strains were isolated from patients cured of their trypanosomiasis by melarsoprol treatment and appear to be heterozygous, containing both a *TbAQP2* gene which contains 18 single nucleotide polymorphisms (SNPs) compared to that found in the wild-type *T. b. gambiense* strain STIB930, as well as a chimera of the first 616 bp of *TbAQP2* (with two SNPs), a 41 bp region of *TbAQP3* and the last 282 bp of *TbAQP2* (Pyana Pati et al., 2014; **Figure 1H**). This *TbAQP2-3*_(617-658)_ chimera from the Masi-Manimba focus retains the potential NSA/NPS selectivity region of TbAQP2, providing a potential explanation for the assumed sensitivity of these strains to melarsoprol treatment.

So far, it has not been investigated whether the various chimeras found in these *T. b. gambiense* field isolates are capable of transporting pentamidine or melarsoprol, and further expression studies are necessary. However, in all the lab-derived and field isolates with reduced sensitivity to, or relapse after treatment with, melarsoprol and/or pentamidine *TbAQP2* has been found to be altered in some way. In the isolates from some cured patients the wild-type *TbAQP2* was present, although with a number of SNPs, alongside a *TbAQP2-3* chimera, which retains the unusual selectivity region of *TbAQP2*. At least one of the chimeras, from the lab-derived resistant strain, has been shown to be incapable of transporting pentamidine; and thus TbAQP2 appears to be a determinant of the efficacy of pentamidine and melaminophenyl arsenicals.

## SELECTIVITY FILTER

The organization of the AQP2/AQP3 locus in the various strains so far investigated is shown in **Figure 1**. In many cases *TbAQP2* has been recombined into a chimeric gene that possesses most of the AQP3 selectivity filter. Thus, the potential of the selectivity regions to determine the MPXR has been investigated (Munday et al., 2014). Synthetic genes encoding either the B48 chimera *TbAQP2-3*_(569-841)_ or *TbAQP3* containing the selectivity region of *TbAQP2* were expressed in the *aqp2/aqp3* null line; only for the *TbAQP2-3*_(569-841)_ chimera (which already contains the first part of the selectivity motif, as part of the first 561 bp of *TbAQP2* in the chimeric protein), did introduction of the second *TbAQP2* selectivity region affect sensitivity to cymelarsan, reaching susceptibility to this drug halfway between the *aqp2/aqp3* null and the same line expressing WT TbAQP2, indicating that more residues in the first portion of *TbAQP2* are necessary than just the predicted selectivity region; this conclusion was underscored by the observation that the effect was only apparent for arsenical drug sensitivity and that pentamidine sensitivity was not affected by the change (Munday et al., 2014).

## MODELLING OF TbAQP2

**FIGURE 2:**
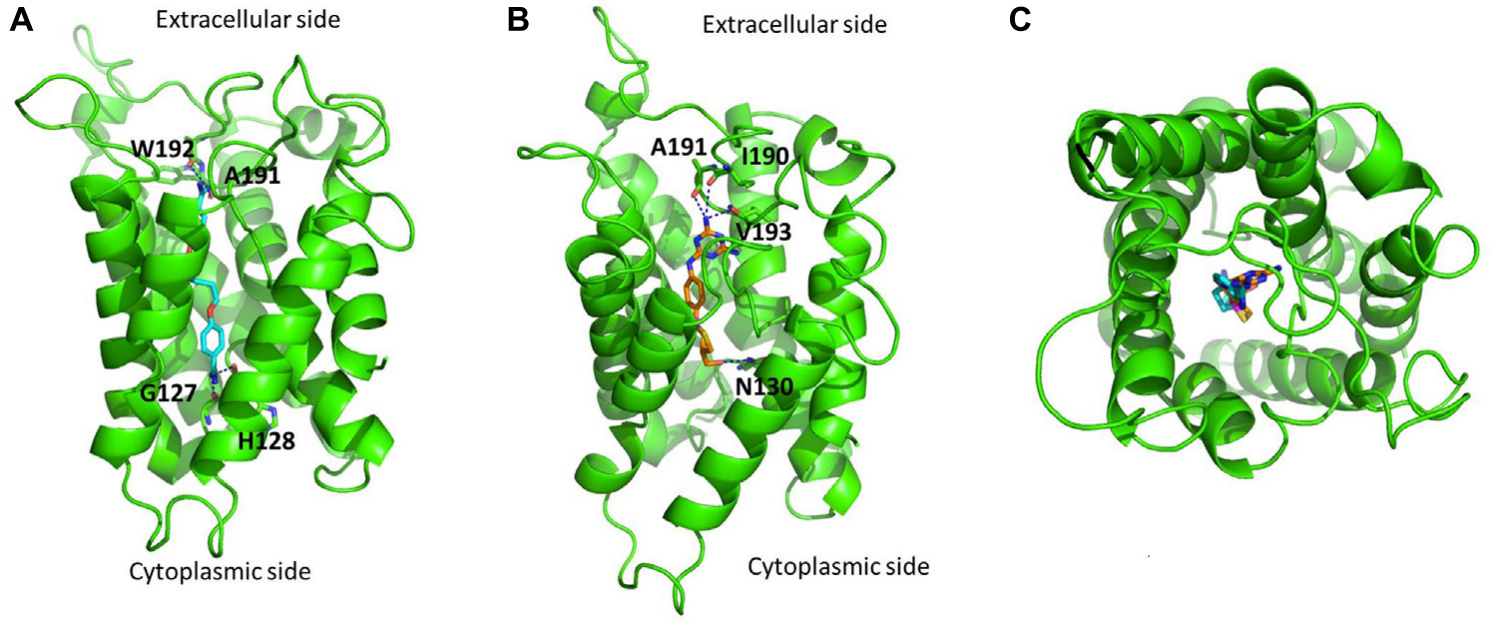
**Predicted binding of pentamidine and melarsoprol in complex with a single TbAQP2 subunit (green). (A)** Binding of pentamidine (cyan carbon atoms). **(B)** Binding of melarsoprol (orange carbon atoms). Key polar interactions are shown for both **(A,B)**. **(C)** Top extracellular view of the overlay of the docked binding poses of pentamidine (cyan carbon atoms) and melarsoprol (orange carbon atoms). The protein was modeled using MODELLER 9.14 (Sali and Blundell, 1993), using as a template the crystal structure of PfAQP (PDB code: 3c02) published by Newby et al. (2008). The sequence identity between TbAQP2 and the template was 33%. The images were created using PyMOL version 1.50.04 (Schrödinger). PyMOL was used to generate the biological units for the aquaglyceroporin from Plasmodium falciparum (generation of symmetry mates). Molecular docking was performed using FRED (McGann, 2012), using a multiconformer database generated using OMEGA. The docked poses were energy minimized using SZYBKI (version 1.7.0) allowing partial relaxation of the protein residues in the direct proximity to the ligand. FRED, OMEGA, and SZYBKI are software developed by OPENEYE (OpenEye Scientific Software: Santa Fe, NM, USA).

The predicted binding modes of pentamidine and melarsoprol are shown in **Figures 2A,B**, respectively. The two guanidine groups of pentamidine are predicted to interact with main-chain carbonyl oxygen atoms of residues located near both the extracellular and cytoplasmic side of the protein channel (**Figure 2A**). Similarly to pentamidine, one of the melamine amino substituents of melarsoprol is predicted to interact with main-chain carbonyl oxygen atoms of residues located near the extracellular side, whilst the hydroxyl group of melarsoprol is predicted to interact with the amidic side-chain group of Asn130 (**Figure 2B**). The pore size of TbAQP2 is sufficiently large to accommodate either pentamidine or melarsoprol (**Figure 2C**), with both ligands able to assume distended conformations.

**FIGURE 3:**
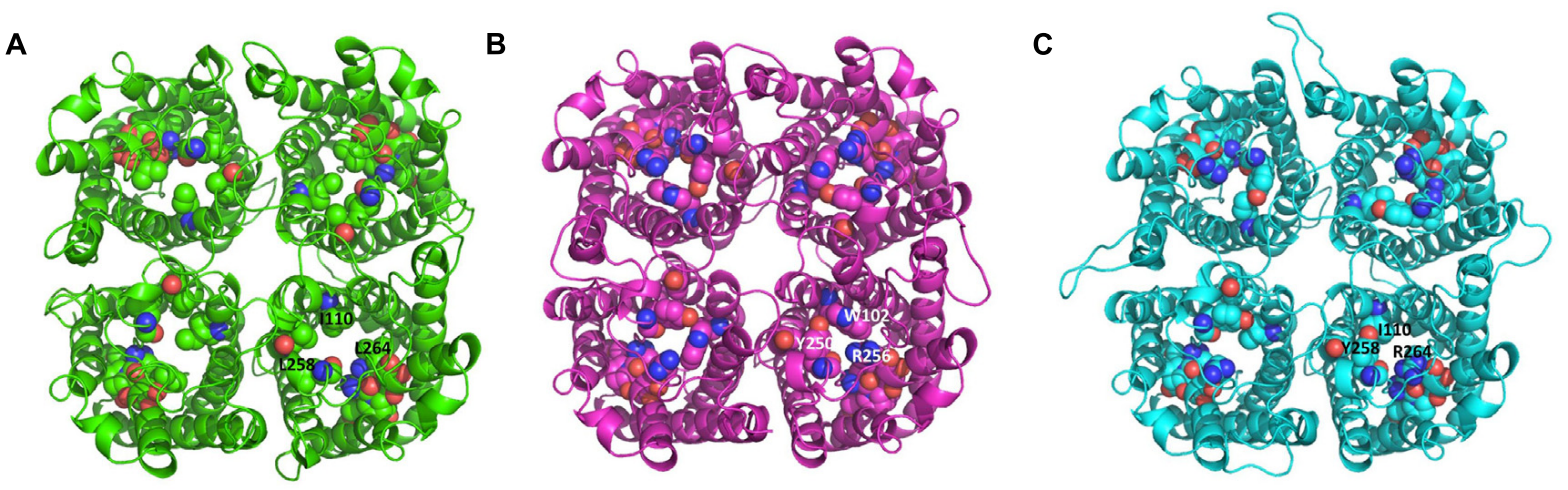
**Extracellular top view of the tetrameric structures. (A)** TbAQP2; the residues constituting the NSA, NPS, and IVLL motif are shown in space-filling models and the locations of Ile110, Leu258, and Leu 264 are indicated in one subunit. **(B)** TbAQP3; the residues constituting the two NPA and WGYR motif are shown in space-filling models and the locations of Trp102, Tyr250, and Arg256 are indicated in one subunit. **(C)** TbAQP2-3_(569-841)_; the residues constituting the NSA, NPS, and IGYR motif are shown in space-filling models and the locations of Ile110, Tyr258, and Arg264 are indicated in one subunit. The images were created using PyMOL version 1.50.04 (Schrödinger). The proteins were modeled using MODELLER 9.14 (Sali and Blundell, 1993), using as a template the crystal structure of PfAQP (PDB code: 3c02; (Newby et al., 2008)). The sequence identity between the target and the template was 33, 36, and 34% for TbAQP2, TbAQP3, and the TbAQP2-3_(569-841)_ chimera, respectively. The C-alpha atoms of chain A, B, C, and D of the tetramer template were restrained during homology modeling using MODELLER in order to reduce the number of interatomic distances that need to be calculated.

The tetrameric structure of the protein models of TbAQP2, TbAQP3, and *TbAQP2-3*_(569-841)_ chimera are shown in **Figures 3A,C**, respectively. The key pore forming residues listed in **Table 1** are also shown in **Figure 3** in space-filling models. The structural alignment of these protein models suggests that TbAQP3 and the TbAQP2-3_(569-841)_ chimera contain pore-forming residues which have bulkier side-chains than with TbAQP2. This could explain the lack of drug transport activity by TbAQP3 and TbAQP2-3_(569-841)_ chimera given the predicted smaller pore in these subunits in comparison with TbAQP2. AQP3, at least, is known to retain normal transport functions for the much smaller water and glycerol substrates (Bassarak et al., 2011), as well as for inorganic As(III) and Sb(III) (Uzcátegui et al., 2013). In particular, TbAQP3 contains three residues (Trp102, Tyr250, and Arg256) whose side-chains protrude into the pore of the channel. These residues align with residues with smaller side-chains in TbAQP2 (Ile110, Leu258 and Leu264, respectively; **Table 1**; **Figures 3A–C**). Similarly, in the TbAQP2-3_(569-841)_ chimera, Tyr258 and Arg264 are likely to be responsible for the lack of transport, as the side-chains of these residues are also predicted to protrude into the pore channel. These predictions are currently under experimental investigation using site-directed mutagenesis.

**Table 1:** Key pore-forming residue differences among TbAQP2, TbAQP3, and the TbAQP2-3_**(569**-**841)**_ chimera.

TbAQP2	TbAQP3	TbAQP2-3_(569-841)_
Ile110	Trp102	Ile110
Asn130	Asn122	Asn130
Ser131	Pro123	Ser131
Ala132	Ala124	Ala132
Val249	Gly241	Gly249
Leu258	Tyr250	Tyr258
Asn261	Asn253	Asn261
Pro262	Pro254	Pro262
Ser263	Ala255	Ala263
Leu264	Arg256	Arg264

## CONCLUDING REMARKS

All the drugs currently in use against trypanosomiasis were identified through *in vivo* disease models and/or phenotypic screens *in vitro*. To a large extent, these toxic chemicals, including diamidines, arsenicals, eflornithine, and even suramin (Delespaux and de Koning, 2007; Alsford et al., 2012, 2013), act selectively on trypanosomes because of unique transport mechanisms, explaining the enormous differences in potency against the closely related *Leishmania* species. Just the expression of the single gene *TbAQP2* in *L. mexicana* promastigotes rendered these parasites 40-fold sensitive to pentamidine and >1000-fold more sensitive to Cymelarsan (Munday et al., 2014). Conversely, the same transporters that make the current chemotherapy against sleeping sickness possible are the cause of drug resistance when their activities are lost.

Thus, the main reason for the sensitivity of trypanosomes to the drugs used against them is that they have unique transporters, and although these transporters are easily recognized as being from ubiquitous gene families, there is currently no way to predict their unusual substrate specificity and role from their primary sequences. The drug transporters so far identified still function efficiently as would be expected from their homologs in other species: i.e., as a purine transporter (P2/TbAT1), an aquaglyceroporin (TbAQP2), and as an amino acid transporter (TbAAT6). It is only because trypanosomal drug transport has been studied in the amount of detail that it has been, that the drug transport activities have been identified for these otherwise relatively unremarkable members of ubiquitous gene families. This leads to the important conclusion that any transporter may be a potential drug transporter and that there is no narrowly defined class of ‘drug transporters,’ nor any transporter families that can be excluded from that category.

As there are so many transporters in the *T. brucei* genome it is possible to do this the other way round, i.e., to try to generate a drug that will enter trypanosomes through a specific transporter (Barrett and Gilbert, 2006; Vodnala et al., 2013). In such a scenario, given that loss of transporters has been demonstrated to give rise to resistance, it would be important to assess by how many transporters any new compounds are taken up; this could lead to minimisation of the risk of transporter-related resistance for new compounds, especially if the transporter, like any individual purine transporter, TbAQP2 or TbAAT6, are non-essential. In each case a single point mutation (such as the introduction of a STOP codon or frame shift) could be sufficient to induce a high level of drug resistance, apparently with no fitness cost.

The unusual aquaglyceroporin TbAQP2 was found to encode the high affinity pentamidine transporter (HAPT1), (Munday et al., 2014), and appears to be vitally important for sensitivity to pentamidine and melaminophenyl arsenicals (Baker et al., 2012; Graf et al., 2013). It may be possible to test for MPXR by assessing the presence of wild-type TbAQP2 alleles in clinical samples. However, this is unlikely to deliver a simple test as there is no single, easily confirmed mutation and the emerging data suggest that many different mutations or rearrangements can give rise to loss of AQP2 as a drug transporter (Graf et al., 2013; Munday et al., 2014; Pyana Pati et al., 2014), it would probably require sequencing of the complete AQP2-AQP3 locus to identify any changes and adjust drug treatment plans accordingly. Even then, we need to know more about the link between observed mutations to the drug transport activity of the gene product, which will require functional expression and analysis of all chimeras and mutants identified. Only if we can gain sufficient insights of all the structural determinants of drug tranport by TbAQP2 may we be able to base treatment decisions on *TbAQP2* sequnce analysis. The model presented here provides testable hypotheses for not just the structure of the aquaporin, but also the residues involved in binding of pentamidine and melarsoprol.

## Conflict of Interest Statement

The authors declare that the research was conducted in the absence of any commercial or financial relationships that could be construed as a potential conflict of interest.

## Footnotes

1 As multiple different chimeric AQP2/3 chimeric genes are being reported in connection with (potential) drug resistance, it is important to have a nomenclature that unambiguously identifies each isolate. In brackets the first numbers indicate the first nucleotide that is unambiguously AQP3 sequence; the second number is the first nucleotide after that to be unambiguously AQP2 sequence. If there is no second number the sequence reads as AQP3 to the end.

## References

[B1] Alsford S. Eckert S. Baker N. Glover L. Sanchez-Flores A. Leung K. F. (2012). High-throughput decoding of antitrypanosomal drug efficacy and resistance. *Nature* 482 232–236. 22278056 10.1038/nature10771 PMC3303116

[B2] Alsford S. Field M.C. Horn D. (2013). Receptor-mediated endocytosis for drug delivery in African trypanosomes: fulfilling Paul Ehrlich’s vision of chemotherapy. *Trends Parasitol.* 29 207–212 10.1016/j.pt.2013.03.004 23601931

[B3] Baker N. Alsford S. Horn D. (2011). Genome-wide RNAi screens in African trypanosomes identify the nifurtimox activator NTR and the eflornithine transporter AAT6. *Mol. Biochem. Parasitol.* 176 55–57 10.1016/j.molbiopara.2010.11.010 21093499 PMC3032052

[B4] Baker N. de Koning H.P. Mäser P. Horn D. (2013). Drug resistance in African trypanosomiasis: the melarsoprol and pentamidine story. *Trends Parasitol.* 29 110–118 10.1016/j.pt.2012.12.005 23375541 PMC3831158

[B5] Baker N. Glover L. Munday J. C. Aguinaga Andres D. Barrett M. P. de Koning H. P. (2012). Aquaglyceroporin 2 controls susceptibility to melarsoprol and pentamidine in African trypanosomes. *Proc. Natl. Acad. Sci. U.S.A.* 109 10996–11001 10.1073/pnas.1202885109 22711816 PMC3390834

[B6] Barrett M.P. Gilbert I. H. (2006). Targeting of toxic compounds to the trypanosome’s interior. *Adv. Parasitol.* 63 125–183 10.1016/S0065-308X(06)63002-9 17134653

[B7] Barrett M.P. Zhang Z. Q. Denise H. Giroud C. Baltz T. (1995). A diamidine-resistant *Trypanosoma* equiperdum clone contains a P2 purine transporter with reduced substrate affinity. *Mol. Biochem. Parasitol.* 73 223–229 10.1016/0166-6851(95)00120-P 8577330

[B8] Bassarak B. Uzcátegui N.L. Schonfeld C. Duszenko M. (2011). Functional characterization of three aquaglyceroporins from *Trypanosoma brucei* in osmoregulation and glycerol transport. *Cell. Physiol. Biochem.* 27 411–420 10.1159/000327968 21471730

[B9] Beitz E. Wu B. Holm L.M. Schultz J. E. Zeuthen T. (2006). Point mutations in the aromatic/arginine region in aquaporin 1 allow passage of urea, glycerol, ammonia, and protons. *Proc. Natl. Acad. Sci. U.S.A.* 103 269–274 10.1073/pnas.0507225103 16407156 PMC1326162

[B10] Bray P. G. Barrett M. P. Ward S. A. de Koning H. P. (2003). Pentamidine uptake and resistance in pathogenic protozoa: past, present and future. *Trends Parasitol.* 19 232–239 10.1016/S1471-4922(03)00069-2 12763430

[B11] Bridges D.J. Gould M. K. Nerima B. Mäser P. Burchmore R.J. de Koning H. P. (2007). Loss of the high-affinity pentamidine transporter is responsible for high levels of cross-resistance between arsenical and diamidine drugs in African trypanosomes. *Mol. Pharmacol.* 71 1098–1108 10.1124/mol.106.031351 17234896

[B12] Brun R. Blum J. Chappuis F. Burri C. (2010). Human African trypanosomiasis. *Lancet* 375 148–159 10.1016/S0140-6736(09)60829-1 19833383

[B13] Brun R. Schumacher R. Schmid C. Kunz C. Burri C. (2001). The phenomenon of treatment failures in human African Trypanosomiasis. *Trop. Med. Int. Health* 6 906–914 10.1046/j.1365-3156.2001.00775.x 11703845

[B14] Carter N.S. Barrett M. P. de Koning H. P. (1999). A drug resistance determinant in *Trypanosoma brucei* *Trends Microbiol.* 7 469–471 10.1016/S0966-842X(99)01643-1 10603477

[B15] Carter N.S. Berger B. J. Fairlamb A. H.(1995). Uptake of diamidine drugs by the P2 nucleoside transporter in melarsen-sensitive and -resistant *Trypanosoma brucei brucei*. *J. Biol. Chem.* 270 28153–28157 10.1074/jbc.270.47.28153 7499305

[B16] Carter N. S. Fairlamb A. H. (1993). Arsenical-resistant trypanosomes lack an unusual adenosine transporter. *Nature* 361 173–176 10.1038/361173a0 8421523

[B17] Cherenet T. Sani R.A. Speybroeck N. Panandam J. M. Nadzr S. Van den Bossche P. (2006). A comparative longitudinal study of bovine trypanosomiasis in tsetse-free and tsetse-infested zones of the Amhara Region, northwest Ethiopia. *Vet. Parasitol.* 140 251–258 10.1016/j.vetpar.2006.04.004 16675127

[B18] Collar C.J. Al-Salabi M. I. Stewart M. L. Barrett M. P. Wilson W. D. de Koning H. P. (2009). Predictive computational models of substrate binding by a nucleoside transporter. *J. Biol. Chem.* 284 34028–34035 10.1074/jbc.M109.049726 19808668 PMC2797173

[B19] Damper D. Patton C. L. (1976). Pentamidine transport and sensitivity in brucei-group trypanosomes. *J. Protozool.* 23 349–356 10.1111/j.1550-7408.1976.tb03787.x 6797

[B20] de Koning H. P. (2001a). Transporters in African trypanosomes: role in drug action and resistance. *Int. J. Parasitol.* 31 512–522 10.1016/S0020-7519(01)00167-9 11334936

[B21] de Koning H. P. (2001b). Uptake of pentamidine in *Trypanosoma brucei brucei* is mediated by three distinct transporters: implications for cross-resistance with arsenicals. *Mol. Pharmacol.* 59 586–592. 11179454 10.1124/mol.59.3.586

[B22] de Koning H. P. (2008). Ever-increasing complexities of diamidine and arsenical crossresistance in African trypanosomes. *Trends Parasitol.* 24 345–349 10.1016/j.pt.2008.04.006 18599351

[B23] de Koning H. P. Anderson L. F. Stewart M. Burchmore R. J. Wallace L. J. Barrett M. P. (2004). The trypanocide diminazene aceturate is accumulated predominantly through the TbAT1 purine transporter: additional insights on diamidine resistance in african trypanosomes. *Antimicrob. Agents Chemother.* 48 1515–1519 10.1128/AAC.48.5.1515-1519.2004 15105099 PMC400564

[B24] de Koning H.P. Bridges D. J. Burchmore R. J. (2005). Purine and pyrimidine transport in pathogenic protozoa: from biology to therapy. *FEMS Microbiol. Rev.* 29 987–1020 10.1016/j.femsre.2005.03.004 16040150

[B25] de Koning H.P. Jarvis S. M. (1999). Adenosine transporters in bloodstream forms of *Trypanosoma brucei brucei*: substrate recognition motifs and affinity for trypanocidal drugs. *Mol. Pharmacol.* 56 1162–1170. 10570043 10.1124/mol.56.6.1162

[B26] de Koning H.P. Jarvis S. M. (2001). Uptake of pentamidine in *Trypanosoma brucei brucei* is mediated by the P2 adenosine transporter and at least one novel, unrelated transporter. *Acta Trop.* 80 245–250 10.1016/S0001-706X(01)00177-2 11700182

[B27] Delespaux V. de Koning H. P. (2007). Drugs and drug resistance in African trypanosomiasis. *Drug Resist. Updat.* 10 30–50 10.1016/j.drup.2007.02.004 17409013

[B28] Delespaux V. Geysen D. Van den Bossche P. Geerts S. (2008). Molecular tools for the rapid detection of drug resistance in animal trypanosomes. *Trends Parasitol.* 24 236–242 10.1016/j.pt.2008.02.006 18420457

[B29] Desquesnes M. Holzmuller P. Lai D.H. Dargantes A. Lun Z. R. Jittaplapong S. (2013). *Trypanosoma evansi and surra: a review and perspectives on origin, history, distribution, taxonomy, morphology, hosts, and pathogenic effects*. *Biomed. Res. Int.* 2013:194176 10.1155/2013/194176 PMC3760267 24024184

[B30] Fikru R. Goddeeris B. M. Delespaux V. Moti Y. Tadesse A. Bekana M. (2012). Widespread occurrence of *Trypanosoma vivax* in bovines of tsetse- as well as non-tsetse-infested regions of Ethiopia: a reason for concern? *Vet. Parasitol*. 190 355–361 10.1016/j.vetpar.2012.07.010 22858227

[B31] Frommel T.O. Balber A. E. (1987). Flow cytofluorimetric analysis of drug accumulation by multidrug-resistant *Trypanosoma brucei brucei* and *T. b. rhodesiense. Mol. Biochem. Parasitol.* 26 183–191 10.1016/0166-6851(87)90142-3 3431564

[B32] Gadelha C. Holden J.M. Allison H. C. Field M. C. (2011). Specializations in a successful parasite: what makes the bloodstream-form African trypanosome so deadly? *Mol. Biochem. Parasitol*. 179 51–58 10.1016/j.molbiopara.2011.06.006 21763356

[B33] Geerts S. Holmes P. H. (1998). *Drug Management and Parasite Resistance in Bovine Trypanosomiasis in Africa*. Rome: Food and Agriculture Organization, PAAT Technical and Scientific Series.

[B34] Geerts S. Holmes P.H. Eisler M. C. Diall O. (2001). African bovine trypanosomiasis: the problem of drug resistance. *Trends Parasitol.* 17 25–28 10.1016/S1471-4922(00)01827-4 11137737

[B35] Gonzatti M.I. González-Baradat B. Aso P.M. Reyna-Bello A. (2014). “*Trypanosoma* (Duttonella) *vivax* and trypanosomosis in Latin America: Secadera/Huquera/Cacho Hueco,” in *Trypanosomes and Trypanosomiasis*, eds Magez S. Radwanska M. (Vienna: Springer-Verlag), 261–285.

[B36] Graf F. E. Ludin P. Wenzler T. Kaiser M. Brun R. Pyana P. P. (2013). Aquaporin 2 mutations in *Trypanosoma brucei gambiense* field isolates correlate with decreased susceptibility to pentamidine and melarsoprol. *PLoS Negl. Trop. Dis.* 7:e2475 10.1371/journal.pntd.0002475 PMC3794916 24130910

[B37] Legros D. Evans S. Maiso F. Enyaru J.C. Mbulamberi D. (1999). Risk factors for treatment failure after melarsoprol for *Trypanosoma brucei gambiense* trypanosomiasis in Uganda. *Trans. R. Soc. Trop. Med. Hyg.* 93 439–442 10.1016/S0035-9203(99)90151-7 10674099

[B38] Li H. Chen H. Steinbronn C. Wu B. Beitz E. Zeuthen T. (2011). Enhancement of proton conductance by mutations of the selectivity filter of aquaporin-1. *J. Mol. Biol.* 407 607–620 10.1016/j.jmb.2011.01.036 21277313

[B39] Lun Z.R. Lai D. H. Li F. J. Lukes J. Ayala F. J. (2010). *Trypanosoma brucei*: two steps to spread out from Africa. *Trends Parasitol.* 26 424–427 10.1016/j.pt.2010.05.007 20561822

[B40] Mäser P. Sutterlin C. Kralli A. Kaminsky R. (1999). A nucleoside transporter from *Trypanosoma brucei* involved in drug resistance. *Science* 285 242–244 10.1126/science.285.5425.242 10398598

[B41] Mathieu C. Gonzalez Salgado A. Wirdnam C. Meier S. Suter Grotemeyer M. Inbar E. (2014). *Trypanosoma brucei* eflornithine transporter AAT6 is a low affinity, low selective transporter for neutral amino acids. *Biochem. J.* 463 9–18 10.1042/BJ20140719 24988048

[B42] Matovu E. Geiser F. Schneider V. Mäser P. Enyaru J. C. Kaminsky R. (2001). Genetic variants of the TbAT1 adenosine transporter from African trypanosomes in relapse infections following melarsoprol therapy. *Mol. Biochem. Parasitol.* 117 73–81 10.1016/S0166-6851(01)00332-2 11551633

[B43] Matovu E. Stewart M.L. Geiser F. Brun R. Mäser P. Wallace L. J. (2003). Mechanisms of arsenical and diamidine uptake and resistance in *Trypanosoma brucei*. *Eukaryot. Cell* 2 1003–1008 10.1128/EC.2.5.1003-1008.2003 14555482 PMC219364

[B44] McGann M. (2012). FRED and HYBRID docking performance on standardized datasets. *J. Comput. Aided Mol. Des.* 26 897–906 10.1007/s10822-012-9584-8 22669221

[B45] Mogk S. Meiwes A. Shtopel S. Schraermeyer U. Lazarus M. Kubata B. (2014). Cyclical appearance of African trypanosomes in the cerebrospinal fluid: new insights in how trypanosomes enter the CNS. *PLoS ONE* 9:e91372 10.1371/journal.pone.0091372 PMC3950183 24618708

[B46] Moore A. Richer M. (2001). Re-emergence of epidemic sleeping sickness in southern Sudan. *Trop. Med. Int. Health* 6 342–347 10.1046/j.1365-3156.2001.00714.x 11348529

[B47] Mumba Ngoyi D. Lejon V. Pyana P. Boelaert M. Ilunga M. Menten J. (2010). How to shorten patient follow-up after treatment for *Trypanosoma brucei gambiense* sleeping sickness. *J. Infect. Dis.* 201 453–463 10.1086/649917 20047500

[B48] Munday J. C. Eze A. A. Baker N. Glover L. Clucas C. Aguinaga Andres D. (2014). *Trypanosoma brucei* aquaglyceroporin 2 is a high-affinity transporter for pentamidine and melaminophenyl arsenic drugs and the main genetic determinant of resistance to these drugs. *J. Antimicrob. Chemother.* 69 651–663 10.1093/jac/dkt442 24235095 PMC3922157

[B49] Munday J. C. Rojas Lopez K. E. Eze A. A. Delespaux V. Van Den Abbeele J. Rowan T. (2013). Functional expression of TcoAT1 reveals it to be a P1-type nucleoside transporter with no capacity for diminazene uptake. *Int. J. Parasitol. Drugs Drug Resist.* 3 69–76 10.1016/j.ijpddr.2013.01.004 24533295 PMC3862423

[B50] Namangala B. Odongo S. (2014). “Animal African trypanosomosis in sub-Saharan Africa and beyond African borders,” in *Trypanosomes and Trypanosomiasis*, eds Magez S. Radwanska M. (Vienna: Springer-Verlag), 239–260.

[B51] Newby Z.E. O’Connell J. III Robles-Colmenares Y. Khademi S. Miercke L.J. Stroud R. M. (2008). Crystal structure of the aquaglyceroporin PfAQP from the malarial parasite *Plasmodium falciparum*. *Nat. Struct. Mol. Biol.* 15 619–625 10.1038/nsmb.1431 18500352 PMC2568999

[B52] Paindavoine P. Pays E. Laurent M. Geltmeyer Y. Le Ray D. Mehlitz D. (1986). The use of DNA hybridization and numerical taxonomy in determining relationships between *Trypanosoma brucei* stocks and subspecies. *Parasitology* 92(Pt 1), 31–50 10.1017/S0031182000063435 3960593

[B53] Priotto G. Kasparian S. Mutombo W. Ngouama D. Ghorashian S. Arnold U. (2009). Nifurtimox-eflornithine combination therapy for second-stage African *Trypanosoma brucei gambiense* trypanosomiasis: a multicentre, randomised, phase III, non-inferiority trial. *Lancet* 374 56–64 10.1016/S0140-6736(09)61117-X 19559476

[B54] Pyana Pati P. Van Reet N. Mumba Ngoyi D. Ngay Lukusa I. Karhemere Bin Shamamba S. Büscher P. (2014). Melarsoprol sensitivity profile of *Trypanosoma brucei gambiense* isolates from cured and relapsed sleeping sickness patients from the democratic republic of the Congo. *PLoS Negl. Trop. Dis.* 8:e3212 10.1371/journal.pntd.0003212 PMC4183442 25275572

[B55] Rambow J. Rönfeldt D. Wu B. Beitz E. (2014). Aquaporins with anion/monocarboxylate permeability: mechanisms, relevance for pathogen-host interactions. *Front. Pharmacol.* 5:199 10.3389/fphar.2014.00199 PMC4150397 25225485

[B56] Robays J. Nyamowala G. Sese C. Betu Ku Mesu Kande V. Lutumba P. Van der Veken W. (2008). High failure rates of melarsoprol for sleeping sickness, Democratic Republic of Congo. *Emerg. Infect. Dis.* 14 966–967 10.3201/eid1406.071266 18507916 PMC2600279

[B57] Rollo I.M. Williamson J. (1951). Acquired resistance to ‘Melarsen,’ tryparsamide and amidines in pathogenic trypanosomes after treatment with ‘Melarsen’ alone. *Nature* 167 147–148 10.1038/167147a0 14806401

[B58] Sali A. Blundell T. L. (1993). Comparative protein modelling by satisfaction of spatial restraints. *J. Mol. Biol.* 234 779–815 10.1006/jmbi.1993.1626 8254673

[B59] Schumann Burkard G. Jutzi P. Roditi I. (2011). Genome-wide RNAi screens in bloodstream form trypanosomes identify drug transporters. *Mol. Biochem. Parasitol.* 175 91–94 10.1016/j.molbiopara.2010.09.002 20851719

[B60] Simarro P.P. Franco J. Diarra A. Postigo J. A. Jannin J. (2012). Update on field use of the available drugs for the chemotherapy of human African trypanosomiasis. *Parasitology* 139 842–846 10.1017/S0031182012000169 22309684

[B61] Song J. Mak E. Wu B. Beitz E. (2014). Parasite aquaporins: current developments in drug facilitation and resistance. *Biochim. Biophys. Acta* 1840 1566–1573 10.1016/j.bbagen.2013.10.014 24140393

[B62] Stanghellini A. Josenando T. (2001). The situation of sleeping sickness in Angola: a calamity. *Trop. Med. Int. Health* 6 330–334 10.1046/j.1365-3156.2001.00724.x 11348527

[B63] Steverding D. (2008). The history of African trypanosomiasis. *Parasit. Vectors* 1:3 10.1186/1756-3305-1-3 PMC2270819 18275594

[B64] Swallow B. M. (2000). *Impacts of Trypanosomiasis on African Agriculture.* Rome: Food and Agriculture Organization, PAAT Technical and Scientific Series.

[B65] Teka I. A. Kazibwe A. J. El-Sabbagh N. Al-Salabi M. I. Ward C. P. Eze A. A. (2011). The diamidine diminazene aceturate is a substrate for the high-affinity pentamidine transporter: implications for the development of high resistance levels in trypanosomes. *Mol. Pharmacol.* 80 110–116 10.1124/mol.111.071555 21436312 PMC3127539

[B66] Uzcátegui N. L. Figarella K. Bassarak B. Meza N. W. Mukhopadhyay R. Ramirez J. L. (2013). *Trypanosoma brucei* aquaglyceroporins facilitate the uptake of arsenite and antimonite in a pH dependent way. *Cell. Physiol. Biochem.* 32 880–888 10.1159/000354490 24217645

[B67] Uzcátegui N. L. Szallies A. Pavlovic-Djuranovic S. Palmada M. Figarella K. Boehmer C. (2004). Cloning, heterologous expression, and characterization of three aquaglyceroporins from *Trypanosoma brucei*. *J. Biol. Chem.* 279 42669–42676 10.1074/jbc.M404518200 15294911

[B68] Vincent I. M. Creek D. Watson D. G. Kamleh M. A. Woods D. J. Wong P. E. (2010). A molecular mechanism for eflornithine resistance in African trypanosomes. *PLoS Pathog.* 6:e1001204 10.1371/journal.ppat.1001204 PMC2991269 21124824

[B69] Vodnala S. K. Lundback T. Yeheskieli E. Sjoberg B. Gustavsson A. L. Svensson R. (2013). Structure-activity relationships of synthetic cordycepin analogues as experimental therapeutics for African trypanosomiasis. *J. Med. Chem.* 56 9861–9873 10.1021/jm401530a 24283924

[B70] Ward C. P. Wong P. E. Burchmore R. J. de Koning H. P. Barrett M. P. (2011). Trypanocidal furamidine analogues: influence of pyridine nitrogens on trypanocidal activity, transport kinetics, and resistance patterns. *Antimicrob. Agents Chemother.* 55 2352–2361 10.1128/AAC.01551-10 21402852 PMC3088251

[B71] WHO. (2009). *WHO Model List of Essential Medicines, 16th List March 2009*. World Health Organization, Geneva Available at: http://www.who.int/medicines/publications/essentialmedicines/en/index.html

[B72] Zeuthen T. Wu B. Pavlovic-Djuranovic S. Holm L. M. Uzcátegui N. L. Duszenko M. (2006). Ammonia permeability of the aquaglyceroporins from *Plasmodium falciparum*, *Toxoplasma gondii* and *Trypansoma brucei*. *Mol. Microbiol.* 61 1598–1608 10.1111/j.1365-2958.2006.05325.x 16889642

